# A case of thymoma-associated multiorgan autoimmunity including polymyositis and myocarditis

**DOI:** 10.1186/s40792-021-01309-1

**Published:** 2021-10-20

**Authors:** Chihiro Furuta, Motoki Yano, Hiroki Numanami, Masayuki Yamaji, Rumiko Taguchi, Masayuki Haniuda

**Affiliations:** Division of Chest Surgery, Department of Surgery, Aichi Medical University, 1-1 Yazakokarimata, Nagakute, 480-1195 Japan

## Abstract

**Background:**

Polymyositis and myocarditis associated with thymoma are exceptionally rare conditions and usually accompanied by myasthenia gravis (MG) and have been recognized as critical conditions. Thymoma-associated multiorgan autoimmunity was reported recently with skin, liver, and intestinal manifestations similar to those seen in graft-versus-host disease.

**Case presentation:**

A 77-year-old female presented to our department with exacerbation of ptosis and local recurrence of thymoma. Chest computed tomography revealed local recurrence of thymoma. Following 6 month observation, erythema on the extremities and body trunk suddenly appeared. Afterwards, the patient developed progressive muscle weakness and fatigue. We diagnosed as myocarditis and polymyositis. She was transferred to the intensive-care unit and received artificial ventilation. Steroid pulse therapy was induced immediately. The blood test findings were markedly improved, but the symptoms of MG and weakness of the muscles persisted. Various treatment including eculizumab was induced, and the symptoms of MG and weakness of the muscles were improved. On the 136th day of hospitalization, she was discharged.

**Conclusion:**

We were able to cure this patient, as we were able to start treatment immediately after the appearance of severe symptoms. An early diagnosis and treatment are important for curing such patients.

## Background

Polymyositis and myocarditis associated with thymoma are exceptionally rare conditions and usually accompanied by myasthenia gravis (MG). Since the first report of four autopsy cases, polymyositis and myocarditis associated with thymoma have gradually come to be recognized as critical conditions [1, 2]. Thymoma-associated multiorgan autoimmunity (TAMA) was reported recently with skin, liver, and intestinal manifestations similar to those seen in graft-versus-host disease (GVHD) [3]. We herein report a case with issues associated with multiple autoimmune diseases, including MG, GVHD-like erythroderma, polymyositis, and myocarditis.

## Case presentation

A 77-year-old female presented to our department with exacerbation of ptosis and local recurrence of thymoma. Five years earlier, she had been diagnosed with an anterior mediastinal tumor with a symptom of ptosis and underwent thymectomy for type B2 thymoma of Masaoka stage II disease. Following the operation, her ptosis was well controlled with tacrolimus 3 mg/day, prednisolone 5 mg/day, and pyridostigmine 180 mg/day.

**Fig. 1:**
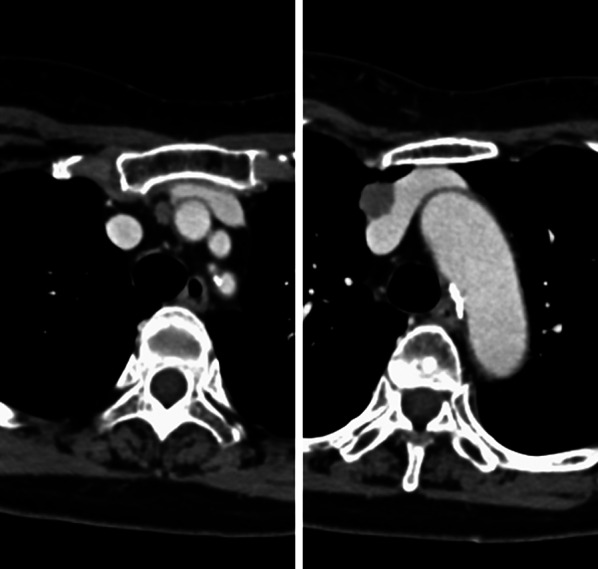
Chest computed tomography (CT) revealed a recurrent lesion in the anterior mediastinum

When she underwent an operation for colon cancer, chest computed tomography as a preoperative examination revealed local recurrence of a nodule 1 cm in diameter on the innominate vein and a small nodule in the anterior mediastinum (Fig. 1). We recommended radiotherapy for the recurrent lesions, but she wished to undergo observation for a while. She hoped to receive treatment for the exacerbation of ptosis.

**Fig. 2:**
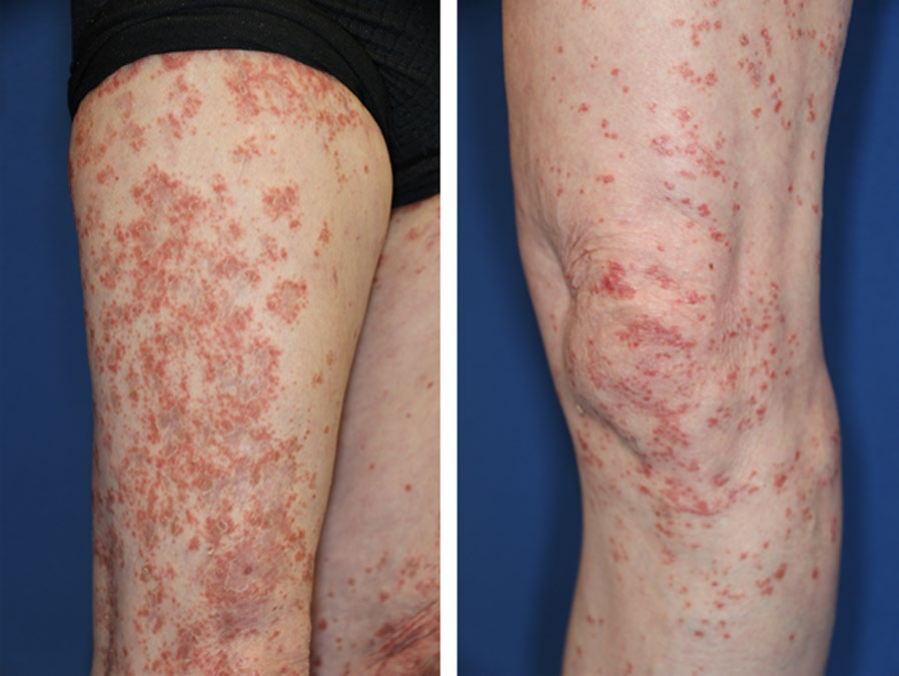
GVHD-like erythroderma. Erythema appeared on the extremities and body trunk

The dose of tacrolimus was increased according to the trough value of the blood concentration, and her ptosis improved. The value of anti-acetylcholine receptor (AChR) antibody gradually decreased (71 to 10 nmol/L). Following 6 month observation, erythema on the extremities and body trunk suddenly appeared (Fig. 2), which was diagnosed as drug eruption. The drugs for MG were thus reduced or interrupted. The patient developed progressive muscle weakness and fatigue and was admitted to the hospital.

**Fig. 3:**
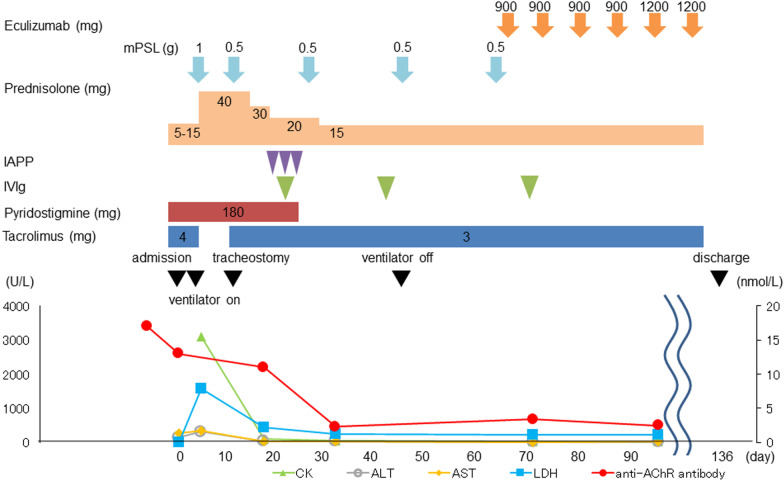
Post-administration course and blood examination values. Muscle weakness did not recover quickly, even after the abnormal values had normalized. Respiration assistance was continued. *mPSL* methylprednisolone, *IAPP* immunoadsorption plasmapheresis, *IVIg* intravenous immunoglobulin, *ICU* intensive-care unit, *CK* creatine kinase, *ALT* alanine aminotransferase, *AST* aspartate aminotransferase, *LDH* lactate dehydrogenase, *anti-AChR antibody* anti-acetylcholine receptor antibody

The post-administration course and blood examination values are shown in Fig. 3. Aspartate aminotransferase (AST) and alanine aminotransferase (ALT) levels were significantly increased, and complication of drug-induced or autoimmune hepatitis was suspected. She slept in the sitting position, because her dyspnea worsened in the supine position. Her neck hung down without bulbar palsy symptoms. We once experienced a similar situation in an MG patient who died of myocarditis and polymyositis. She was transferred to the intensive-care unit (ICU) 5 days after admission and received artificial ventilation.

A laboratory examination performed immediately before treatment revealed that the serum levels of creatinine phosphokinase (3098 U/L; normal range 45–163 U/L), creatine kinase isozyme MB (257 U/L; normal range 45–163 U/L), ALT (311 U/L; normal range 6–27 U/L) and AST (351 U/L; normal range 13–33 U/L) were elevated. In addition, cardiac troponin I was elevated to 679.6 pg/mL (normal range 0–26.2 pg/mL). Serum electrolyte and calcium values were normal. The electrocardiogram at admission to the ICU showed only a slightly prolonged QT time (0.392 s). The echocardiogram demonstrated a preserved left ventricular systolic function, with an ejection fraction (EF) of 63%. There were no findings of cardiac infarction. Although a myocardial biopsy could not be performed, we diagnosed as polymyositis and myocarditis which is rarely associated with thymoma. This diagnosis was valid considering the positive findings of anti-striational autoantibodies (anti-titin and anti-Kv1.4), elevation of myosin light chain I and luck of elevation of anti-AChR antibody, which were revealed later.

The clinical course is shown in Fig. 3. Steroid pulse therapy was induced immediately for polymyositis and myocarditis. As the cardiac function was maintained, catecholamine administration was not needed. The blood test findings were markedly improved, as shown in Table 1, but the symptoms of MG and weakness of the muscles persisted. Therefore, we performed tracheostomy and immunoadsorption plasmapheresis (IAPP).

**Table 1:** Changes in blood test data

Blood test (units, normal range)	3 Weeks before admission	Day 1 (admission to the hospital)	Day 6 (admission to the ICU)	Day 9 (7 days after ICU admission)	Day 13 (7 days after ICU admission)
WBC (× 1000/μL, 5.0–8.0)	10.3	13.0	12.1	11.3	9.7
CRP (mg/dL, ≤ 0.3)	2.63	14.7	12.58	2.38	2.51
AST (U/L, 13–33)	33	268	351	67	19
ALT (U/L, 6–27)	19	145	311	208	55
LDH (U/L, 119–229)	292	1015	1587	922	352
CK (U/L, 45–163)	118	–	3098	229	66
CKMB (U/L, ≤ 25)	–	–	257	52	21
BNP (pg/mL, ≤ 18.4)	–	–	92.7	216.2	37.4
Anti-AChR antibody (nmol/L: ≤ 0.2)	23	13	–	–	–

*ICU* intensive-care unit, *WBC* white blood cell counts, *CRP* C-reactive protein, *AST* aspartate aminotransferase, *ALT* alanine aminotransferase, *LDH* lactate dehydrogenase, *CK* creatine kinase, *CKMB* creatine kinase–myoglobin binding, *BNP* brain natriuretic peptide, *anti-AChR antibody* anti-acetylcholine receptor antibody

Pancytopenia and hypogammaglobulinemia (Good's syndrome) were also found, and intravenous immunoglobulin therapy was performed. She was also diagnosed with cytomegalovirus infection, and ganciclovir was administered. Eculizumab was induced, and the symptoms of MG and weakness of the muscles were improved. On the 136th day of hospitalization, she was discharged. Since then, immune therapy with medicine has been continued, excluding eculizumab. There has been no recurrence of any of her autoimmune diseases, excluding MG, for 1 year.

## Discussion

We experienced a case with multiple autoimmune diseases, including MG, GVHD-like erythroderma, polymyositis, myocarditis, hypogammaglobulinemia, and pancytopenia. Thymoma is the most common tumor of the anterior mediastinum. It is often associated with autoimmune diseases. The representative disorder is MG, and thymomas are sometimes detected following the appearance of MG symptom. Complication only with MG has not been considered a poor prognostic factor of thymoma [4]. Meanwhile, other autoimmune diseases, such as pure red cell aplasia (PRCA) and hypogammaglobulinemia, are also widely recognized as being associated with thymoma, and these conditions are often found in patients with advanced disease or recurrence [5]. Treatment of advanced thymoma along with various autoimmune disease can, therefore, be complex.

TAMA is a recently delineated and rare paraneoplastic syndrome reported in patients with thymoma. The first report of TAMA described GVHD-like manifestations in the skin, liver, and intestines [3]. Since then, similar cases have been reported. Shiba et al. reviewed 29 cases of TAMA. Among those patients, 72.4% (21/29) had skin lesions, and 17 of those 21 cases with skin lesions had a fatal course [6]. Complications of TAMA, especially skin lesions, can thus lead to a poor prognosis. In the 29 patients with TAMA, complication with MG or PRCA was recognized, but polymyositis and myocarditis which were not found. However, 2 of the 29 patients died suddenly for no obvious reason. It may be possible that those patients were complicated with myocarditis. The present case showed a variety of symptoms, such as GVHD-like skin lesions, liver dysfunction, polymyositis and myocarditis, MG and PRCA, which were not mentioned in previous reports.

Polymyositis and myocarditis associated with thymoma are exceptionally rare and usually accompanied by MG [1, 2]. These entities are diagnosed in approximately 1% of patients with thymoma and are characterized by acute heart failure, ventricular arrhythmias, or heart block that rapidly develops a fatal course, usually within 10 days [7]. Notably, some cases do show a good response to steroid, azathioprine, and cyclosporine therapy. However, Tanahashi et al. reported that glucocorticoid pulse therapies were not effective [8]. Delayed detection of this disease may reduce the effectiveness of treatment. The present case was the second such case we had ever experienced [9]. Unfortunately, the first patient died before treatment could be administered, because we were unfamiliar with this disease and took a long time to diagnose it. The diagnosis of myocarditis associated with is not easy without performing an autopsy. This time, however, we were able to start steroid pulse therapy immediately after the diagnosis before the cardiac function had deteriorated. It took some time for the patient to recover her muscle strength due to the complication with MG, but she eventually recovered with a good course.

We administered eculizumab to the present patient. Eculizumab is a humanised monoclonal antibody that specifically binds with high affinity to human terminal complement protein C5. Recently, the REGAIN study showed that eculizumab was effective and well tolerated in patients with anti-acetylcholine receptor antibody-positive refractory generalized MG (gMG). Oyama et al. reported that patients with refractory gMG with myasthenia crisis and thymoma-associated MG were suitable for eculizumab administration. Whether or not eculizumab was effective in the present patient is unclear, but it can be expected to be effective against thymoma-associated MG and polymyositis and myocarditis, as both diseases are caused by autoimmune antibodies.

## Conclusions

We experienced a rare case with multiple autoimmune diseases, including TAMA, polymyositis, and myocarditis. Fortunately, we could successfully treat this patient, as we were able to start treatment immediately after the appearance of severe symptoms. An early diagnosis and treatment are important for curing such patients.

## Data Availability

All data supporting this article are including in the published article.

## Abbreviations

MG: Myasthenia gravis
TAMA: Thymoma-associated multiorgan autoimmunity
GVHD: Graft-versus-host disease
AChR: Anti-acetylcholine receptor
AST: Aspartate aminotransferase
ALT: Alanine aminotransferase
IAPP: Immunoadsorption plasmapheresis
PRCA: Pure red cell aplasia
